# Population-based cross-sectional study of factors influencing full vaccination status of children aged 12- 23 months in a rural district of the Upper East Region, Ghana

**DOI:** 10.1186/s12887-024-04662-w

**Published:** 2024-03-08

**Authors:** Emmanuel Awonanya Akanpaabadai, Abraham Awonboro Adiak, Ruth Nimota Nukpezah, Martin Nyaaba Adokiya, Simon Effah Adjei, Michael Boah

**Affiliations:** 1 Ghana Health Service, Private Mail Bag (PMB), Bolgatanga, Ghana; 2 Tamale Teaching Hospital, P.O. Box TL 16, Tamale, Ghana; 3 School of Nursing and Midwifery University for Development Studies, Northern Region, Tamale, Ghana; 4 Department of Epidemiology, Biostatistics, and Disease Control, School of Public Health, University for Development Studies, Tamale, Ghana; 5 Center for Population Health, Institute of Global Health Equity Research, University of Global Health Equity, Kigali, Rwanda

**Keywords:** Children, Ghana, Rural, Sub-Saharan Africa, Vaccination, Population-based, Immunisation

## Abstract

**Background:**

Achieving universal health coverage includes ensuring that children have access to vaccines that are of high quality, safe, efficacious, and affordable. The Immunisation Agenda 2030 aims to expand services to zero-dose and incompletely vaccinated children and reduce immunisation rate disparities as a contribution to vaccination equity. This study explored the factors influencing full vaccination status among children aged 12 – 23 months in a rural district of the Upper East Region of Ghana.

**Methods:**

A population-based cross-sectional study was conducted among carers of children aged 12 -23 months in the Kassena Nankana West district. A multistage sampling technique was used to select 360 carers. Information regarding the vaccination status of children was gathered through a combination of children’s health record books and carers’ recollections. Information on potential determinants was also systematically collected for analysis in Stata version 15.0.

**Results:**

The results showed that 76.9% (95% CI: 72.3 – 81.0) of children had full vaccinations per the national schedule. All children received at least one vaccination. A higher percentage of carers with incompletely vaccinated children reported that they had travelled with their children as the primary reason for missing certain vaccine doses. Full vaccination status was significantly associated with secondary (aOR = 2.60; 95% CI: 1.20—5.63) and tertiary (aOR = 3.98, 95% CI: 1.34—11.84) maternal educational level, being in a partnership relationship (aOR = 2.09, 95% CI: 1.03—4.25), and residing in close proximity to healthcare facilities (aOR = 0.41, 95% CI: 0.21—0.80).

**Conclusions:**

Our study found that nearly one-quarter of children aged 12—23 months in the study setting are underserved with vaccination services for a variety of reasons. Effectively reaching these children will require strengthening health systems, including eliminating vaccine shortages, addressing the unique challenges faced by unmarried women with children aged 12–23 months, and improving accessibility to vaccination services.

## Background

Vaccination protects children from preventable diseases and promotes overall public health. Since 2021, immunisation, due to vaccine administration, is estimated to avert nearly 5 million deaths a year [1]. Deaths caused by vaccine-preventable diseases (VPD) disproportionately affect low- and middle-income countries (LMICs). In August 2020, the Seventy-Third World Health Assembly endorsed the Immunisation Agenda 2030 (IA2030) as a global strategy to leave no one behind. The IA2030 defines what needs to happen to achieve its vision of a world where everyone, everywhere, at every age, fully benefits from vaccines for good health and well-being by leaving no one behind, in any situation, or at any stage of life [2]. Additionally, a Framework for Action was developed with four critical elements, namely coordinated planning, monitoring and evaluation, ownership and accountability, and communications and advocacy [3]. This Framework for Action describes how each of the four critical elements will be integrated to ensure the successful implementation of the IA2030 strategy and realise the IA2030 vision.

The coverage of three doses of diphtheria-tetanus-pertussis-containing vaccine (DTP) at age 12 months, an indicator often used to assess countries performance in providing routine immunisation services to children, experienced a decline in global coverage [4]. Children who have not received any doses of DTP by age 12 months (zero-dose children) represent those with poor access to immunisation and other essential health services [5]. Children who receive the first DTP dose (DTP1) but do not complete the full series are considered incompletely vaccinated. Globally, DPT3 coverage decreased from an average of 86% during 2015–2019 to 83% in 2020 and 84% in 2022 [5, 6]. The decline in vaccination coverage during 2020–2021 was likely a result of many factors, including strained health systems caused by the coronavirus disease of 2019 (COVID-19) pandemic [7]. These strains have led to challenges with supply chains, human resources, and financing. Additionally, the rise in vaccine misinformation, disinformation, and hesitancy is likely a contributing factor to the declines experienced in certain countries [8–10]. Furthermore, coverage during 2019–2021 declined more sharply in countries that transitioned out of Global Alliance for Vaccines and Immunisations (GAVI) support than in those supported by GAVI [11]. To reverse declining vaccination rates, fill in gaps in immunity, and build on previous gains in vaccination coverage above levels seen before the pandemic, targeted and situation-specific approaches are needed.

Since 2000, GAVI has assisted LMICs by supporting vaccines and vaccination services. This aid has been essential in improving access and reducing gaps in vaccination coverage between LMICS and high-income countries. Though there has been tremendous progress in immunisation efforts, the vaccination coverage rates in many LMICs, particularly in rural areas of sub-Saharan Africa (SSA), continue to be below the desired level [12]. Ghana, which is located in West Africa, is also faced with this challenge [13]. The country has used the Expanded Programme on Immunisation (EPI), which offers vaccinations against several VPDs, since 1978. These diseases include measles, polio, tuberculosis, and diphtheria. The EPI started with six antigens, and it gradually expanded the number of antigens on its schedule. The current routine childhood vaccines consist of Bacillus Calmette-Guérin (BCG), HepB, Oral Polio Vaccine (OPV), Inactivated Polio Vaccine (IPV), pentavalent or DPT–HepB–Hib vaccine (diphtheria, pertussis, tetanus; hepatitis B; and Haemophilus influenzae type B), pneumococcal conjugate vaccine (PCV), rotavirus vaccine (RV), measles-rubella (MR) vaccine, yellow fever vaccine, and meningococcal A vaccine in Ghana [14].

In Ghana’s historical context, two indicators of vaccination coverage have been used. One of the indicators is the percentage of children receiving all “basic” antigens, and the other indicator is the percentage of children aged 12–23 months and 24–35 months who have received all the recommended vaccines per the national vaccination schedule. To have received all basic antigens, a child in Ghana must receive at least one dose of the BCG vaccine, three doses of both polio and DPT-containing vaccines, and one dose of MR. The percentage of children in Ghana who have received all basic antigens has declined recently compared to more than a decade ago. The preliminary findings from the latest Ghana Demographic and Health Survey (GDHS) indicate that, in 2022, 73% of children aged 12–23 months were fully vaccinated with basic antigens [15]. This is a decline from the 79% recorded in 2008. Similarly, according to the same report, 56% of children aged 12–23 months were fully vaccinated according to the national schedule in the same year. The report further highlighted that the benefits of vaccination are not equally distributed, exhibiting disparities between rural and urban areas. In 2022, 50% of rural children in Ghana were fully vaccinated, while urban children had a higher rate of 62% [15]. Prior studies have shown that factors like maternal age, marital status, employment, ethnicity, and religion have an impact on children’s vaccination status in urban areas of Ghana [16, 17]. To provide universal access to vaccination services for all children, it is crucial to understand the factors that influence vaccination status, particularly in rural settings. This understanding is essential for designing focused interventions to improve vaccination coverage.

The purpose of the present study was to explore the factors that influence vaccination status among children aged 12–23 months in the Kassena Nankana West (KNW) district, a predominantly rural district of the Upper East Region of Ghana.

## Methods

### Study setting

The study was conducted in the KNW district, which was created in 2008 from the Kassena-Nankana Municipality in Ghana’s Upper East Region. The district shares borders with neighbouring districts, including Builsa North, and Kassena-Nankana Municipality, and Burkina Faso, within the Guinea Savannah woodland [18]. The population of the district, according to the 2021 population and housing census, stands at 90,735, nearly evenly split between males and females. About 90% of the population resides in rural areas, living in sparsely settled compounds, posing challenges for healthcare delivery. Healthcare facilities include a hospital, health centres (*n* = 5), private clinics (*n* = 2), and community-based health planning and services (CHPS) compounds (*n* = 32). In total, there are forty (40) health facilities in the district. The climate exhibits two main seasons, including a short rainy season from June to September with an average rainfall of 850–1000 mm (https://www.ghanadistricts.com/Home/District/155). Farming is the primary occupation, with crops essential to local diets and animal husbandry playing significant roles.

### Study design

A population-based cross-sectional study design was adopted to conduct this study [19]. Information on the vaccines children had taken and the potential determinants was obtained from the respondents simultaneously. The information was collected from respondents at their homes.

### Sample and sample size determination

This study specifically targeted mothers or carers of children aged 12–23 months living in the study setting. The study sample was calculated using the single population formula, with a design effect of 1.2 to account for differences within and between clusters. We assumed a coverage of full vaccination of 77% [14]. Furthermore, we assumed a 5% margin of error with a 95% confidence level, corresponding to a Z-statistic of 1.96. In total, 360 respondents were required for the current study, accounting for a 10% non-response rate.

### Sampling method

A multistage sampling technique was adopted to select clusters and households. The cluster sampling technique was used for the selection of clusters (enumeration areas) and households as recommended by the World Health Organisation (WHO) for vaccine coverage surveys [20]. The district has 115 communities (clusters). A simple random sampling technique was used to select thirty (30) enumeration areas, and a proportionate allocation method was utilised to determine the number of households to be selected in each cluster for study. Mother–child pairs were selected systematically from households in each cluster. In the cluster, the starting point was selected by spinning a pen at the midpoint of the community, and the direction the pen faced was followed for the first household. Then, the next household nearest to the selected one was visited first. Only one eligible child in the household was considered for the study. Where a selected household did not have an eligible child, the next nearest household to that household was visited. This process was repeated until the number of eligible children in each cluster was reached. In cases involving twins or triplets, the index child in the household was identified and selected for the study.

### Data collection procedures

Information was collected electronically from participants in face-to-face interviews at their homes using a structured questionnaire programmed onto Kobo Collect Toolbox software installed on smartphones running Android operating systems. The questionnaire included questions about socio-demographic attributes, factors affecting healthcare accessibility and utilisation, questions aimed at assessing carers’ knowledge regarding vaccination, and information about the child’s vaccination. Three field enumerators were recruited and trained on the study protocol and questionnaires. The training involved translating the questions into Kassem and Nankam, the two predominant languages in the study area. Furthermore, an initial questionnaire pretest was conducted at a Kassena Nankana Municipality health facility. This assessment involved 35 carers who had children aged 12–23 months. This pretest aimed to determine the suitability of the data collection tool for gathering the required information and to evaluate the field enumerators’ understanding and use of the tool.

Information regarding vaccination status was gathered from carers using two methods. Firstly, the maternal and child health record book was reviewed, and details of administered vaccines and their respective vaccination dates were extracted. In cases where the immunisation record indicated that the child had not received all the necessary vaccines, the mother or carer was asked to confirm whether the child had received additional vaccines not recorded in the booklet. If the maternal and child’s health record book was unavailable, the data collectors relied on the carer’s recall. These recalls included the location of vaccine administration and the precise dates and methods of administration. Studies have shown that maternal recollection is effective in accurately identifying vaccinated children between the ages of 12 and 23 months in African communities, with a sensitivity comparable to that of information obtained from vaccination cards [21–23]. The presence of a scar on the skin above the insertion point of the deltoid muscle on the upper right arm served as corroborating evidence of BCG vaccine administration. This location is designated as the recommended site for BCG vaccination in Ghana. Respondents were asked to provide the primary reasons why their children had not received a complete vaccination. The study collected data throughout September 2021.

### Variables

#### Dependent variable

The dependent variable, the child’s immunisation status, was assessed by determining whether the child had taken all the antigens expected for their age according to the EPI schedule in Ghana. The outcome of interest was complete or full vaccination; a child aged 12–23 months completed vaccination if there was evidence (by card, observation, or history) of administration of all antigens in the EPI schedule. Specifically, the child has had all the basic antigens, as well as an OPV vaccine at birth, an IPV dose, three doses of HepB and Hib (as part of a vaccine with DPT), three doses of pneumococcal vaccine, two doses of rotavirus vaccine, and one dose of yellow fever vaccine [15]. A child was defined as “not vaccinated” if there was no evidence from the card, observation, or questioning that they had received any vaccination in the Ghanaian immunisation schedule. It was incompletely vaccinated if any one dose was missing for age. No child was observed not to have taken any of the antigens. As a result, a binary dependent variable (completely or fully vaccinated = 1 and incompletely vaccinated = 0) was used for the inferential analysis.

#### Independent variables

Utilising a multidimensional framework, explored a range of potential determinants, including sociodemographic factors such as the child’s sex, maternal age, level of education, ethnicity, religion, parental knowledge about immunisation, healthcare use, and access factors such as antenatal care (ANC) use, place of delivery, and distance to the nearest health facility. These determinants have been widely documented in the literature as influencing vaccination uptake in various settings [12, 13, 24, 25].

Carers’ knowledge about vaccination was assessed using seven questions with predetermined answers, including the benefits of immunisation, whether diseases can be prevented through vaccination, vaccine-preventable diseases, and childhood vaccines. Scores were given for both correct and incorrect responses. A carer scored 1 point for a correct response and 0 points for an incorrect response. The scores were summed, and the average score was computed. A binary variable for knowledge was then created using the average score as the cutoff. A carer who scored above the average was classified as having high knowledge and low knowledge if otherwise. This method of assessing knowledge was adapted from the existing literature [26–28].

### Data processing and analysis

The collected information was downloaded in Excel file format and imported into STATA 15.0/IC for Windows (StataCorp LLC, 4905 Lakeway Drive, College Station, TX, 77845, USA) for cleaning and analysis. Descriptive statistics such as frequencies and percentages were used to examine the distribution of carers by socio-demographic, healthcare utilisation, and access-related factors, as well as the children who had complete vaccination status. A chi-square test was used to perform bivariate analysis to examine associations between independent variables and the binary outcome variable, vaccination status. A multivariable logistic regression was built to assess the association between the study outcome and all other variables considered to be significantly associated with the study outcome at the bivariate level. The final model was interpreted using adjusted odds ratios (aORs) and corresponding 95% confidence intervals. A *p*-value of less than 0.05 was the criterion for statistical significance. The Hosmer–Lemeshow goodness-of-fit test for the multiple logistic regression model was used to assess the fit of our final model in identifying the factors associated with complete vaccination coverage among children [29].

### Ethical issues

For this study, ethical approval was sought from the University of Health and Allied Sciences Research Ethics Committee. Also, written approval was obtained from the District Director of Health Services of the Kassena-Nankana District. Written informed consent was obtained from the respondents before they were interviewed. The study was conducted based on the ethical issues outlined in the Declaration of Helsinki [30]. At the height of COVID-19, respondents and the research team observed adherence to the Ministry of Health's COVID-19 prevention guidelines during the data collection process, which primarily involved face-to-face interviews. These include observing physical distance and wearing nose masks at all times during the interviews. Face masks were distributed to respondents who had none and were encouraged to wear them before the interviews.

## Results

### Background characteristics of the respondents

The socio-demographic characteristics of the respondents, including their children, are presented in Table 1. The distribution of the children by sex was almost equal. However, a higher proportion (94.4%) were not enrolled in school during the survey. A higher proportion (95.3%) of the carers were the biological parents of the index children. Among the respondents, 54.4% were aged 25–34 years of age, 54.4% were employed, and 83.3% were in a union (i.e., married or cohabiting). The results also showed that about 32.0% of the carers independently decided where to seek care for their sick children. The majority of the carers exhibited a high level of knowledge regarding immunisation (56.0%), and 88.0% were able to receive the necessary vaccinations. More than 8 out of 10 respondents perceived the distance to the nearest health facility as a minor problem (83.9%).

**Table 1:** Background characteristics of caregivers and children in the study (*N* = 360)

Variable name	Frequency	Percentage
Sex of Child		
Female	179	49.7
Male	181	50.3
Child enrolled in school		
No	340	94.4
Yes	20	5.6
Respondent is the biological parent of the child		
No	17	4.7
Yes	343	95.3
Age group of caregivers		
15–24	106	29.4
25–34	196	54.4
35 +	58	16.2
Level of education		
No formal education	68	18.9
Primary	123	34.2
Secondary	110	30.5
Tertiary	59	16.4
Employment status		
Unemployed	164	45.6
Employed	196	54.4
Marital status		
Not married	60	16.7
In a union (i.e., married or cohabiting)	300	83.3
Religion		
African Traditional	19	5.3
Christian	254	70.6
Muslim	87	24.1
Caregiver's ethnicity		
Kassena	244	67.8
Nankam	111	30.8
Others	5	1.4
Decision maker on where to seek care when child is sick		
Husband/Partner (Father)	239	66.4
Mother (Self)	114	31.7
Others	7	1.9
Antenatal care visits		
No visits	2	0.6
Less than 4	58	16.1
4 and above	300	83.3
Place of delivery		
Home	11	3.1
Health facility	349	96.9
Attendant at birth		
None	5	1.4
Traditional birth attendant (TBA)	13	3.6
Health worker (Nurse/midwife/Doctor)	342	95.0
Level of knowledge about immunisation		
Low	159	44.2
High	201	55.8
Able to receive the needed service at vaccination sessions		
Able to receive	317	88.1
Not able to receive	43	11.9
Perception about health providers' responsiveness		
Not Sure	23	6.4
Unprofessional	13	3.6
Very Professional	324	90.0
Time to reach the nearest health facility		
Less than 30 min	188	52.2
30—60 min	153	42.5
1 h or more	19	5.3
Problem with distance to the nearest health facility		
Not a big problem	302	83.9
A big problem	58	16.1

### Vaccination status of children 12 – 23 months in the study setting

**Fig. 1:**
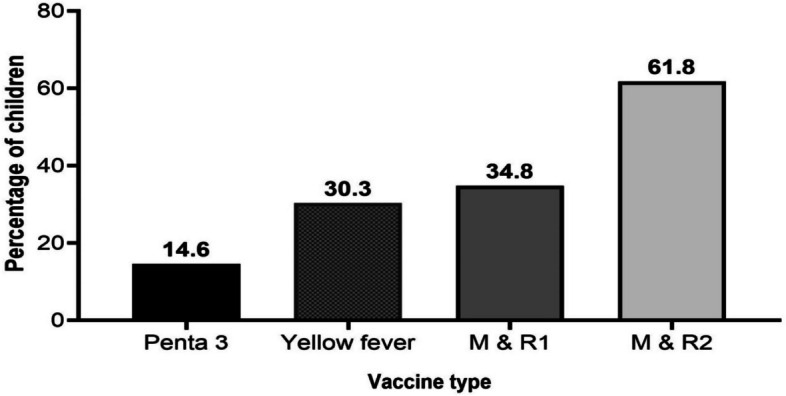
Vaccines missed by children 12–23 months who had incomplete vaccinations (*N* = 83). M & R: Measles & Rubella vaccine; Penta: pentavalent vaccine

Of the 360 children, 76.9% (95% CI: 72.3 – 81.0) were found to have a complete vaccination for their age. None of the children were unvaccinated. Figure 1 presents data regarding the vaccines that were commonly missed among children with incomplete vaccinations for their age. Among this subgroup, the highest percentage of children (61.8%) missed the second dose of the measles and rubella vaccine, with the third dose of the pentavalent vaccine (diphtheria, tetanus, pertussis, hepatitis B, and Haemophilus influenzae type B) being the least missed vaccine (14.6%).

### Main reasons mentioned by carers for children missing vaccination

Table 2 provides an overview of reasons given by carers for their children not being fully vaccinated. Among the carers surveyed (*N* = 83), the most common reason was travelling with the child (28.9%). Subsequently, some carers (15.7%) reported being uninformed about their child’s immunisation schedule, while others cited being busy (15.7%) as the reason. In addition, 8.4% of carers stated that they had to change the scheduled time for vaccinations because of the unavailability of vaccines or not having enough children to use a whole vaccine vial. About 7.0% indicated that they were ignorant of their child’s incomplete immunisation status. Other reasons included the child being sick, not having a weighing card, being at a remote location like a farm in the bush, the child being in school, and the distance to the health facility. Each of these reasons accounted for smaller proportions of the responses, ranging from 1.2% to 3.6%.

**Table 2:** Main reasons given by caregivers for incomplete vaccination of their children (*N* = 83)

Reasons	Frequency	Percentage
Travelled with child	24	28.9
I am not aware child is due	13	15.7
I was busy	13	15.7
Forgot to take child	8	9.6
Rescheduled (vaccine not available or number of children not many enough to open vaccine)	7	8.4
Not aware child is not fully vaccinated	6	7.2
Child was sick	3	3.6
Child does not have weighing card	3	3.6
Was at farm in the bush	3	3.6
Child is in School	1	1.2
Distance to health facility	1	1.2
Others	1	1.2

### Association between child vaccination status and child-, maternal-, and contextual-level factors

The study employed the chi-square test to investigate the association between child vaccination status and various exposure variables. Results revealed statistically significant associations between the vaccination status of children and several variables. Notably, carers’ level of education (*p* = 0.003), employment status (*p* = 0.007), marital status (*p* = 0.005), religious affiliation (*p* = 0.042), and ethnicity (*p* = 0.002) revealed significant relationships with vaccination status. Specifically, a higher proportion of carers with tertiary education (89.8%), those employed (82.7%), those in unions (79.7%), and those practicing Islam (83.9%) had their children fully vaccinated compared to their respective counterparts (Table 3).

**Table 3:** Association between child vaccination status and child-, maternal-, and contextual-level factors

Variable	Frequency	Child vaccination Status		*p-*value
		Perecentage (%) of incompletely vaccinated	Percentage (%)fully vaccinated	
Sex of Child				0.350
Female	179	25.1	74.9	
Male	181	21.0	79.0	
Child enrolled in school				0.738
No	340	23.2	76.8	
Yes	20	20.0	80.0	
Respondent is the biological parent of the child				0.220
No	17	35.3	64.7	
Yes	343	22.4	77.6	
Age group of caregiver				0.227
15–24	106	27.4	72.6	
25–34	196	23.0	77.0	
35 +	58	15.5	84.5	
Level of education				0.002
No formal education	68	36.8	63.2	
Primary	123	26.0	74.0	
Secondary	110	18.2	81.8	
Tertiary	59	10.2	89.8	
Employment status				0.005
Unemployed	164	29.9	70.1	
Employed	196	17.3	82.7	
Marital status				0.006
Not married	60	36.7	63.3	
In a union (i.e., married or cohabiting)	300	20.3	79.7	
Religion				0.041
African traditional	19	42.1	57.9	
Christian	254	24.0	76.0	
Muslim	87	16.1	83.9	
Decision-maker on where to seek care when child is sick				0.032^a^
Husband/partner	239	20.1	79.9	
Mother or carer (Self)	114	27.2	72.8	
Others	7	57.1	42.9	
Antenatal care visits				0.005^a^
No visits	2	50.0	50.0	
Less than 4	58	37.9	62.1	
4 and above	300	20.0	80.0	
Place of delivery				0.136^a^
Home	11	45.5	54.5	
Health facility	349	22.3	77.7	
Attendant at birth				0.469^a^
None	5	0.0	100.0	
Traditional birth attendant	13	30.8	69.2	
Health worker (Nurse/midwife/doctor)	342	23.1	76.9	
Level of knowledge about immunisation				0.274
Low	159	25.8	74.2	
High	201	20.9	79.1	
Able to receive the needed service at vaccination sessions				0.019
Able to receive	317	21.1	78.9	
Not able to receive	43	37.2	62.8	
Perception about health providers' responsiveness				0.160^a^
Not Sure	23	39.1	60.9	
Unprofessional	13	15.4	84.6	
Very Professional	324	22.2	77.8	
Time to reach the nearest health facility				0.328
Less than 30 min	188	22.9	77.1	
30—60 min	153	21.6	78.4	
1 h or more	19	36.8	63.2	
Problem with distance to the nearest health facility				0.001
Not a big problem	302	19.9	80.1	
A big problem	58	39.7	60.3	

^a^*p* – values from Fisher’s exact test

Furthermore, higher rates of complete vaccination were seen among carers whose partners made all decisions about where to take their sick children (79.9%), those who went to at least four ANC visits before giving birth (80%), and those who were able to get the necessary vaccination services whenever they went to vaccination sessions (79.1%). The results showed that carers who perceived the distance to the nearest health facility as not a big problem demonstrated a higher complete vaccination rate (80.1%) for their children than those who perceived it as a big problem.

### Independent factors influencing the vaccination status of children aged 12–23 months in the study setting

The study employed adjusted logistic regression analysis to explore the independent associations between the exposure variables identified as statistically significant in the chi-square test and the likelihood of achieving full vaccination status. Table 4 presents the adjusted odds ratios, 95% confidence intervals, and corresponding *p*-values for each exposure variable. Notably, a significant association was observed between the carer’s level of education, marital status, proximity to the nearest health facility, and the vaccination status of the children. It was shown that children whose carers had a secondary education had nearly three times higher odds of being fully vaccinated than those with no formal education (aOR = 2.60; 95% CI: 1.20—5.63). Additionally, children whose carers had a tertiary education had even higher odds of being fully vaccinated (aOR = 3.98, 95% CI: 1.34—11.84). Regarding marital status, carers in a union had higher odds for full vaccination than those who were not married (aOR = 2.09; 95% CI: 1.03—4.25). Lastly, carers who perceived the distance to the nearest health facility as a big problem had significantly lower odds of fully vaccinating their children than those who perceived the distance not to be a big problem (aOR = 0.41; 95% CI: 0.21—0.80). Conversely, variables such as employment status, religion, decision-maker on seeking care for a sick child, antenatal visits made by the carer, and the ability to receive necessary services during immunisation sessions did not show statistically significant associations with the vaccination status of children in the adjusted analysis.

**Table 4:** Factors independently associated with the full vaccination status of children aged 12–23 in the study setting (*N* = 360)

Variable	aOR	95% Confidence interval	*p*—value
Level of education			
No formal education	Ref	1.00	
Primary	1.50	0.74—3.05	0.258
Secondary	2.60	1.20—5.63	0.016
Tertiary	3.98	1.34—11.84	0.013
Employment status			
Unemployed	Ref	1.00	
Employed	1.51	0.86—2.65	0.147
Marital status			
Not married	Ref	1.00	
In a union (i.e., married or cohabiting)	2.09	1.03—4.25	0.041
Religion			
African traditional	Ref	1.00	
Christian	0.84	0.27—2.61	0.767
Muslim	1.64	0.48—5.67	0.431
Decision-maker on where to seek care for sick child			
Others	Ref	1.00	
Husband/partner	1.50	0.27—8.24	0.638
Mother/carer	1.11	0.20—6.21	0.904
Antenatal visits			
4 or more	Ref	1.00	
No visits	0.13	0.01—2.18	0.155
Less than 4	0.80	0.39—1.65	0.548
Able to receive the needed service at vaccination sessions			
Not able to receive	Ref	1.00	
Able to receive	2.12	0.97—4.60	0.058
Problem with distance to the nearest health facility			
Not a big problem	Ref	1.00	
A big problem	0.41	0.21—0.80	0.009
Goodness-of-fit test			
Number of observations	360		
number of groups	10		
Hosmer–Lemeshow chi^2^ (8)	10.66		
Prob > chi^2^	0.221		

*aOR* Adjusted odds ratio, *Ref* Reference

The goodness-of-fit test, evaluating the model’s compatibility with the data, yielded a chi-square test statistic value of 10.66 with 8 degrees of freedom and a *p*-value of 0.221, indicating an acceptable fit of the model to the observed data.

## Discussion

In Ghana, vaccination coverage rates vary based on the geographical location and study methodology. The present population-based cross-sectional study investigated the factors influencing the full vaccination status of children aged 12–23 months in one of the districts of the Upper East Region of Ghana. Our study reveals that more than three-quarters of children aged 12–23 months in the rural study setting are fully vaccinated. According to a study conducted in the middle zone of Ghana, nearly 90% of children had full vaccination status [16]. A secondary analysis of national data showed that full vaccination coverage had improved in Ghana since 1998 and was approximately 92% in 2014 [13]. However, the most recent GDHS reported that approximately 56% of children aged 12 -23 months were fully vaccinated in Ghana [15].

The percentage of fully vaccinated children also appears to vary widely between countries in the sub-Saharan Africa subregion. For example, systematic reviews have reported a coverage rate of about 59% in Ethiopia [24] and 63% in Senegal [31]. A multi-level analysis of factors associated with incomplete vaccination status in Togo reported a complete vaccination coverage of about 72%, which is lower than the coverage reported in the current study [32]. In Nigeria, over three-quarters of children have full vaccination status, with coverage being lower in rural communities (56%) compared to urban areas (95%) [33]. In a rural setting in Gambia, two-thirds of children aged 12–23 months are reported to be completely vaccinated [34]. It is worth mentioning that a review of studies in the SSA has demonstrated that coverage is relatively low when vaccination coverage is reported at the national level [8]. Moving beyond the African region, a national study conducted in Indonesia found that 62% of children were fully vaccinated [35]. The low coverage reported by studies in LMICs is an indication that significant efforts are still required to avert the deaths caused by VPDs, particularly in the African region, and to achieve the full health impact of vaccination at the global level [1].

Multiple factors impede the provision of vaccines and access to vaccine-related services in the SSA. While some of these factors pertain to the health care system and its providers, others concern the mothers or carers of children who qualify for vaccines. Health system barriers that impede the delivery of vaccines to children aged 12–23 months include disruptions in the cold chain, inconsistent vaccine supplies and distribution leading to shortages, inadequate infrastructure and human resources, and considerable distances separating healthcare facilities from families [8, 10, 36]. It is important to highlight that providers’ efforts to minimise vaccine wastage also inadvertently create missed opportunities for vaccination [10, 36]. Demand-side factors contributing to the under-vaccination or non-administration of vaccines to children in SSA include financial constraints, prevailing beliefs concerning vaccination, inadequate access to information and knowledge, and carers’ time constraints and unavailability [8, 10, 36]. Consistent with the reasons reported in previous studies, the present study identified several contributing factors to incomplete childhood vaccination, with travel, lack of awareness, and busy schedules being the primary reasons reported by most carers.

The results revealed that the carer’s level of education, marital status, and proximity to the nearest health facility influenced the vaccination status of children. Children whose carers had secondary or tertiary education were more likely to be completely vaccinated compared to those with no formal education. This aligns with previous studies that have shown a relationship between maternal education and vaccination coverage [12, 13, 24, 25]. Educated carers may have better access to health information, understand the benefits of immunisation, and have improved decision-making abilities regarding their children’s healthcare [27, 36]. Additionally, educated women may have a higher socioeconomic status, which can provide them with better access to healthcare facilities and services, including vaccination programmes [8].

The present study found an association between partnership status and child vaccination status, which is consistent with previous studies [16, 17, 26, 37]. In particular, children of carers in a union had a higher likelihood of achieving complete vaccination status compared to those who were not married. A study in rural Mozambique did not report significant differences between carers of children with or without complete vaccination status and marital status [38]. Though the evidence on the association between marital status and child vaccination status is inconsistent in the Mozambique study, other previous studies have associated being in union with a higher likelihood of having a child with a complete vaccination status [17, 26, 37]. According to a study conducted in Kampala, Uganda, many women reported receiving substantial support from their partners to ensure their child’s vaccination [39]. This support primarily included financial assistance for transportation expenses and the granting of permission to facilitate the child’s vaccination process. These results highlight the significant influence of male partners on childhood vaccination, with the degree of influence ultimately depending on whether the male partner holds beliefs that support or oppose childhood vaccination.

The association between perceived proximity to the nearest health facility and child vaccination status is another important finding of the present study. This finding aligns with a previous study that identified geographic accessibility as a significant barrier to providing immunisation services in some SSA countries [12]. Carers who considered the distance to the nearest health facility a big problem were less likely to have their children completely vaccinated, likely due to long travel distances, which can be time-consuming, expensive, and physically demanding [36, 39]. This issue may impede timely access to vaccination services, resulting in lower vaccination rates [8, 10]. This barrier is particularly relevant in rural or underserved areas, where healthcare facilities are often limited or inaccessible. While distance is widely acknowledged in the literature as a significant barrier influencing the receipt of essential health services, other studies have not found significant differences in vaccination status among children due to distance [31].

### Policy interpretation

The present study aligns with previous studies that demonstrate the influence of carers’ education and marital status on vaccination outcomes. Carers with higher education levels and those in unions are more likely to have their children fully vaccinated. Additionally, our study reinforces the importance of addressing geographical barriers to improve vaccination coverage. These findings contribute to the existing body of knowledge and emphasise the need for targeted interventions to increase education levels, enhance marital support, and improve geographical access to healthcare services for optimal vaccination coverage.

### Strengths and limitations

The present study provides evidence for targeted interventions for improving vaccination coverage among children aged 12 – 23 months in the study setting. The sample size is representative of the targeted population. Thus, the findings may be generalised to children within that age group in the study area. The model’s fitness was also evaluated to ensure that the multiple regression model was a good fit for identifying the factors influencing vaccination coverage among children. Nevertheless, there are certain limitations of the present study that have to be acknowledged. Firstly, we used a cross-sectional study design, which limits our conclusions to only associations rather than causal inferences. Furthermore, many known factors in the literature can influence vaccination coverage; however, not all of these factors were accounted for in the present analysis. Moreover, we acknowledge that relying on maternal recall of events predisposes the study to recall bias. Specifically, in the absence of vaccination cards, vaccination status was based on carers’ recall. However, it is important to note that maternal recall of children’s vaccination status has been established to be accurate with minimal recall bias [22].

## Conclusions

Our study found that nearly one-quarter of children aged 12–23 months in the study setting are underserved with vaccination services for various reasons. This underscores the multifaceted nature of the factors influencing full vaccination status among this age group. Maternal education, marital status, and geographical proximity to healthcare facilities are important influencers. Effectively reaching these children requires strengthening health systems, including an adequate supply of vaccines, addressing the unique challenges faced by unmarried women with children aged 12–23 months, and improving accessibility to vaccination services.

## Data Availability

The minimal data supporting this study’s conclusions are found within the publication. The dataset, on the other hand, can be obtained from the corresponding author on reasonable request.

## Abbreviations

ANC: Antenatal care
BCG: Bacillus Calmette-Guérin
CHPS: Community-based health planning and services
COVID-19: Corona virus disease of 2019
DPT–HepB–Hib vaccine: Diphtheria, pertussis, tetanus; hepatitis B; and Haemophilus influenzae type B
DTP: Diphtheria-tetanus-pertussis-containing vaccine
Gavi: Global Alliance for Vaccines and Immunisations
GDHS: Ghana Demographic and Health Survey
HepB: hepatitis B; Hib: Hemophilus influenza B
IA2030: Immunisation Agenda 2030
IPV: Inactivated polio vaccine
KNW: Kassena Nankana West
LMICs: Low-and middle-income countries
Men A: Meningococcal A vaccine
MR: Measles-rubella
OPV: Oral polio vaccine
PCV: Pneumococcal conjugate vaccine
RV: Rotavirus vaccine
WHO: World Health Organisation
